# The feasibility of occupational therapy using Zones of Regulation™ Concepts in an equine environment

**DOI:** 10.3389/fpsyt.2024.1401222

**Published:** 2024-09-03

**Authors:** B. Caitlin Peters, Robin L. Gabriels, Arlene A. Schmid, Zhaoxing Pan, Tamara Merritt, Arielle Hoffman, Susan Hepburn

**Affiliations:** ^1^ Temple Grandin Equine Center, Department of Animal Sciences, Colorado State University, Fort Collins, CO, United States; ^2^ Department of Occupational Therapy, Colorado State University, Fort Collins, CO, United States; ^3^ Department of Psychiatry, University of Colorado Anschutz Medical Campus, Aurora, CO, United States; ^4^ Biostatistics Core of Children’s Hospital Colorado Research Institute, Department of Pediatrics, University of Colorado Anschutz Medical Campus, Aurora, CO, United States; ^5^ Hearts & Horses, Inc. Director of Program Innovation and Research, Loveland, CO, United States; ^6^ Program in Occupational Therapy, Washington University School of Medicine, St. Louis, MO, United States; ^7^ Department of Human Development and Family Studies, Colorado State University, Fort Collins, CO, United States

**Keywords:** occupational therapy, hippotherapy, equine-assisted services, autism, feasibility

## Abstract

**Introduction:**

The current paper aimed to assess the feasibility of a modified intervention protocol named “Occupational Therapy using Zones of Regulation Concepts in an Equine Environment” (OT-ZOR Equine).

**Methods:**

A single arm A-B feasibility study was conducted, involving 14 autistic youth ages 6-13 years who first received 10-weeks of occupational therapy without horses (OT-ZOR Clinic) followed by 10-weeks of OT-ZOR Equine.

**Results:**

All participants completed the study and attended 95% of OT-ZOR Equine sessions. Occupational therapists maintained 91% fidelity to the OT-ZOR Equine intervention protocol and there were no serious adverse events. All participants’ caregivers and study occupational therapists rated being satisfied or very satisfied with the OT-ZOR Equine intervention. Youth demonstrated improved self-regulation following participation in the OT-ZOR Clinic and OT-ZOR Equine interventions. However, participants’ social functioning only improved after OT-ZOR Equine.

**Discussion:**

This study demonstrated that OT-ZOR Equine is feasible to implement, acceptable to recipients and providers, and may offer additive benefits in social functioning compared to occupational therapy intervention without horses. The current study provides a foundation for future efficacy research aimed at quantifying additive benefits of integrating horses into occupational therapy for autistic youth.

## 1 Introduction

Autism is defined by deficits in social communication and by the presence of restricted or repetitive patterns of behavior (1). Individuals on the autism spectrum [referred to henceforth as “autistic” (2)] often have unique strengths, such as strengths in visual perceptual tasks, certain aspects of auditory processing, or above-average performance in specific skills (e.g., memory, reading, drawing, music, etc.) (3, 4). Despite these strengths, many autistic individuals have difficulty with self-regulation, defined as the ability to monitor, evaluate, and modify one’s arousal levels, emotional states, and behavior in order to execute goal-oriented actions (5, 6). Impaired self-regulation is believed to be inherent in autism, as evidenced by difficulty managing emotions (7), heightened physiological reactivity to daily activities (8, 9), and increased irritability, hyperactivity, aggression, elopement, and self-injury (10, 11). Impaired self-regulation in autistic youth can impact their daily living and quality of life, as evidenced by increased anxiety (12), poor social adjustment (13), and decreased academic performance (14).

Human-animal interaction, particularly with horses, appears to improve self-regulation in autistic youth (15). Two reviews of studies of animal-assisted interventions for autistic youth published 2012-2015 (16) and 2016-2020 (17) found that across different types of animal-assisted interventions, studies often found decreased problematic behaviors, increased positive emotions, and decreased physiological and behavioral indicators of stress (16).

One widespread application of human-animal interaction for autistic youth is equine-assisted services, an umbrella term for services that incorporate horses in order to benefit human health and wellbeing (18). There are over 700 centers across the US that provide equine-assisted services, and autism is consistently identified as the population most often served at these centers (19). One type of equine-assisted service, adaptive riding, is a recreational service focused on teaching horsemanship skills to individuals with disabilities; adaptive riding has been demonstrated to improve self-regulation and social outcomes in autistic youth (15). The beneficial effects of interacting with horses in a recreational setting provides a strong foundation for the notion that including horses in therapy services may lead to more efficacious therapy. However, very little is known about the efficacy of integrating horses into occupational therapy for autistic youth (20). Existing research is scarce and lacks standardized intervention protocols (21–24) and active comparison groups (21, 22, 24). This knowledge gap results in scant empirical information to guide occupational therapy and creates barriers to access services, as many payers do not reimburse for occupational therapy that integrates horses. Consequently, there is a critical need to establish how to best integrate horses into occupational therapy for autistic youth, and empirically demonstrate the additive benefit horses may have on therapy outcomes.

To fill this gap, our team embarked upon a program of research focused on developing and empirically evaluating occupational therapy integrating horses for autistic youth. We have previously developed an occupational therapy protocol named OT^ee^ HORSPLAY (Occupational Therapy in an Equine Environment: Harnessing Occupation for Self-regulation, Social Skills, and Play). Further described in a separate paper (25), OTee HORSPLAY integrated best practices in occupational therapy for autistic youth (26) (individualized goals, social skills training, activity-based intervention, integrations of strengths and interests, scaffolding, and multi-sensory activities) with purposeful inclusion of horses in the intervention to optimize youth’s engagement. In particular, intervention development was guided by the hypothesized principles that 1) the movement of the horse, adjusted as needed by the therapist, can help optimize the youth’s physiological arousal and behavioral regulation during the session, 2) interacting with horses can be motivating for many children, enhancing youths’ attention and engagement in the therapy session, and 3) activities with horses can serve as powerful positive reinforcement for attempting new skills.

In a previous feasibility study we demonstrated that OT^ee^ HORSPLAY improved self-regulation and social outcomes in autistic youth (25) and was largely feasible to implement (27), but also identified several areas of further development needed prior to readiness for large-scale efficacy testing. Namely, OT^ee^ HORSPLAY broadly addressed self-regulation skills, social skills, *or* play skills; the priority area of intervention was determined during an occupational therapy evaluation. The broad nature of OT^ee^ HORSPLAY addressing three distinct goal areas led to large variability in the way the intervention was delivered, and therefore variable outcomes among participants. Thus, we determined there was a need for further standardization of the OT^ee^ HORSPLAY intervention protocol to allow for more uniform delivery across occupational therapists, while still allowing for individualization. Furthermore, our previous feasibility study identified a need to develop a feasible control group for the non-animal elements of OT^ee^ HORSPLAY (i.e. occupational therapy without horses).

### 1.1 Modifications to previously manualized intervention protocol

**Figure 1:**
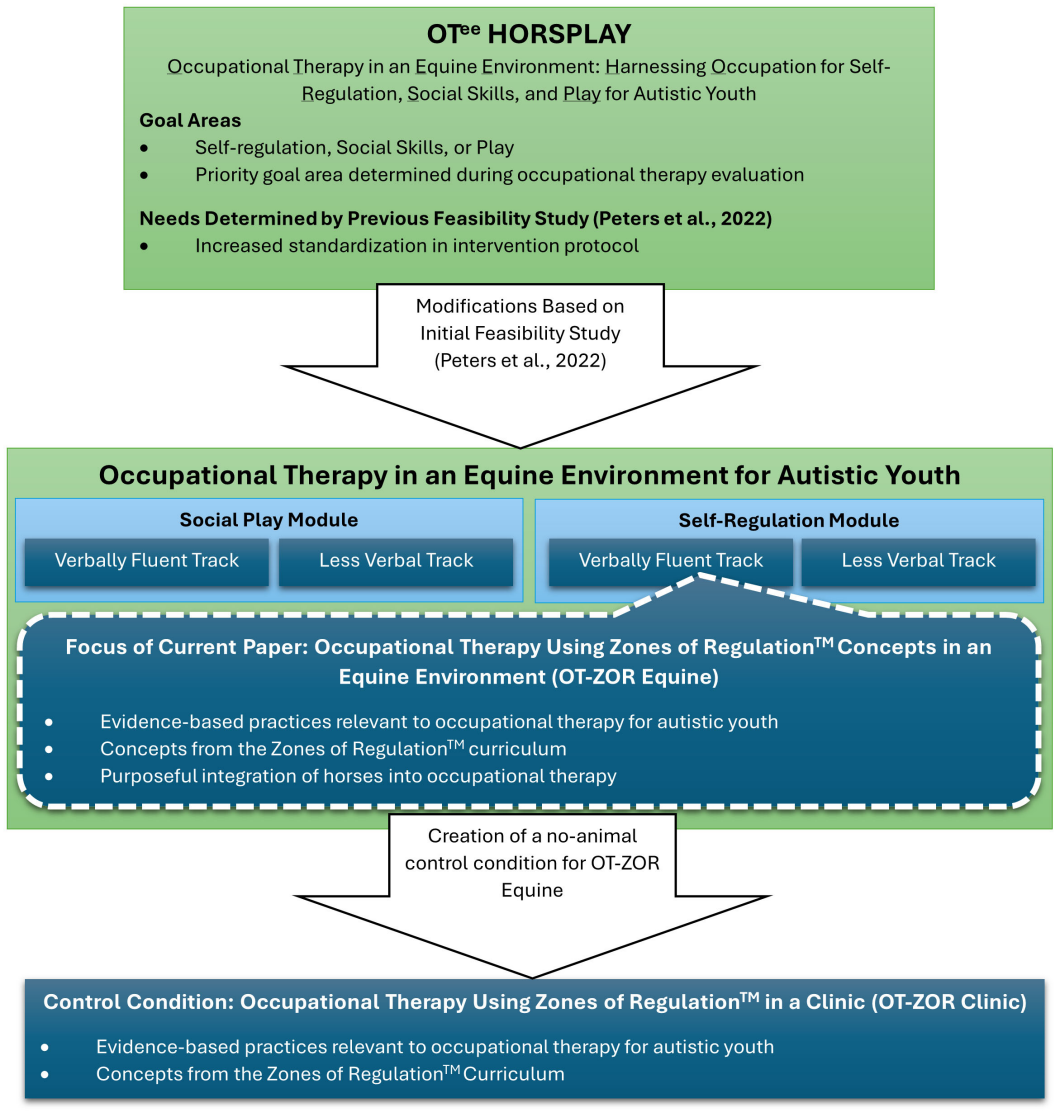
Situation of the current feasibility paper within the larger program of research.

To address these needs, our team made major modifications to the intervention and study protocols, illustrated in **Figure 1**. First, to further standardize the OT^ee^ HORSPLAY manual we created two different modules: social play and self-regulation. Within each module, we also created two tracks: a track for participants with verbally fluent language, and a track for less verbal participants. In the current study, we asked caregivers to select either the self-regulation or social play module; the study team then assigned the child to a track within that module based on verbal fluency. Importantly, most caregivers selected the self-regulation module and were assigned to the verbally fluent track. *Therefore, this paper reports on occupational therapy in an equine environment addressing self-regulation skills in autistic youth who are verbally fluent.*

In creating the self-regulation module of occupational therapy in an equine environment, we chose to integrate concepts from the Zones of Regulation™ curriculum into the intervention manual. Developed by an occupational therapist, the Zones of Regulation™ curriculum teaches youth to recognize emotions in others and in themselves, categorize emotions into 4 different “zones”, and use “tools” to regulate their emotions. The resulting intervention protocol therefore integrates 1) evidence-based practices relevant to occupational therapy for autistic youth, 2) concepts from the Zones of Regulation curriculum, and 3) purposeful integration of horses into occupational therapy. We’ve named this intervention protocol Occupational Therapy using Zones of Regulation in an Equine Environment (OT-ZOR Equine).

Next, our team developed a control condition for all non-animal elements of OT-ZOR Equine, called Occupational Therapy using Zones of Regulation in a Clinic (OT-ZOR Clinic). This control condition combines evidence-based practices in occupational therapy with concepts from the Zones of Regulation curriculum (28).

### 1.2 Purpose and specific aims

Newly developed interventions should be assessed for feasibility prior to being evaluated for efficacy (29). Feasibility studies address the question “Can it work?” considering the following aspects: participant recruitment, intervention implementation and fidelity, intervention acceptability and adherence, data collection procedures, and preliminary participant outcomes (30). During the process of feasibility testing, investigators are encouraged to “refine their intervention through iterative development and then test the feasibility of their final approach” (31). Therefore, after further revisions guided by our previous feasibility study (27), we conducted a new study to assess the feasibility of OT-ZOR Clinic and OT-ZOR Equine. A separate paper reports on the feasibility of the new control condition, OT-ZOR Clinic (28). The current paper’s purpose is to report on the feasibility of the OT-ZOR Equine intervention. Specifically, we aimed to evaluate: 1) participant recruitment, retention, and attendance, 2) intervention fidelity, safety, and caregiver assessment completion, 3) acceptability of the OT-ZOR Equine intervention to providing occupational therapists and caregivers of autistic youth, and 4) preliminary participant outcomes after the OT-ZOR Equine intervention.

## 2 Methods

### 2.1 Design

We implemented a single-arm A-B feasibility study (not randomized), where all youth first participated in ten weeks of OT-ZOR Clinic, immediately followed by 10 weeks of OT-ZOR Equine. Outcome measures were completed at three time points: 1) pre-test, 2) after 10 weeks of OT-ZOR Clinic, and 3) after 10 weeks of OT-ZOR Equine. We also collected feasibility and acceptability data throughout the study. This design allowed us to test the feasibility of both OT-ZOR Clinic and OT-ZOR Equine in all participants, to prepare for a future study focused on efficacy. Peters et al. (28) reports feasibility of the OT-ZOR Clinic condition; this paper reports on the feasibility of the OT-ZOR Equine condition.

### 2.2 Participants

**Figure 2:**
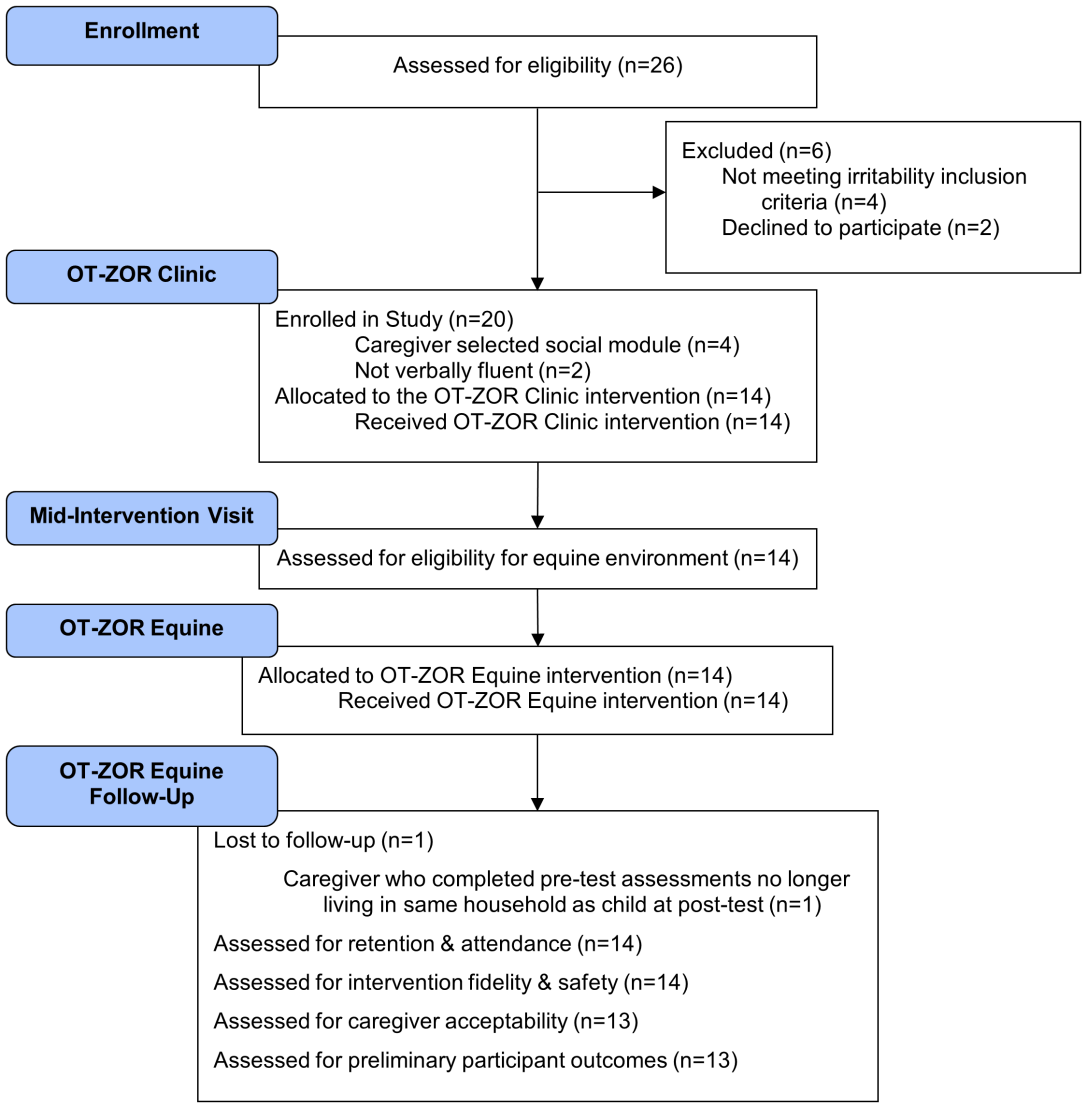
Modified CONSORT Diagram. OT-ZOR Clinic=Occupational Therapy using Zones of Regulation™ Concepts in a Clinic; OT-ZOR Equine=Occupational Therapy using Zones of Regulation™ Concepts in an Equine Environment. Adapted from “CONSORT 2010 Statement: Extension to Randomized Pilot and Feasibility Trials” by S. Eldridge, C. Chan, M. Campbell, C. Bond, S. Hopewell, L. Thabane, and G. Lancaster, 2016, BMJ, 355, p. 20.

We distributed IRB-approved electronic flyers to community organizations to recruit autistic youth and their caregivers. Flyers advertised that youth would participate in both 10 weeks of occupational therapy in a clinic and 10 weeks of occupational therapy in an equine environment. **Table 1** lists participant inclusion and exclusion criteria, and **Figure 2** illustrates participants’ progression through the study. Youth were first screened for inclusion in occupational therapy in a clinic through a two-part process that included 1) an online survey and 2) virtual screening visit using Microsoft Teams due to the Covid-19 pandemic. Twenty autistic youth and their caregivers were enrolled in the study. Consistent with other autism intervention feasibility studies (32–35), we chose a sample of 20 to allow for participant variability on the feasibility indicators defined below. Of the original 20 participants, 14 met additional inclusion criteria for the verbally-fluent self-regulation module (**Figure 1**; **Table 1**) and therefore received OT-ZOR Clinic followed by OT-ZOR Equine, the focus of this paper. After completing 10 weeks of OT-ZOR Clinic, youth were screened for inclusion in the second half of the study by participating in an additional screening visit for occupational therapy in an equine environment (**Table 1**); all youth met criteria and were included in the next 10 weeks of OT-ZOR Equine. This paper therefore focuses on the 14 participants who completed OT-ZOR Clinic and then OT-ZOR Equine.

**Table 1:** Participant inclusion and exclusion criteria.

Original Inclusion Criteria	Original Exclusion Criteria
1. Age 6 – 13 years old 2. Score ≥ 11 on the SCQ 3. Diagnosed with ASD by a community provider 4. Meet clinical cut-offs for ASD on the ADOS on diagnostic report OR confirmed social-communication impairments during virtual screening visit (adaptation made for social distancing) 5. Able to follow 1-step directions 6. Score >10 on the irritability subscale of the ABC-C 7. Meet symptom criterion score on CASI-5 for mood, anxiety, or ADHD diagnosis	1. Rode a horse for 5 hours or more in the last 6 months 2. Weigh more than 200 pounds. 3. Planning on starting any intensive new therapies during the 1-year study period.
Additional Inclusion Criteria for Zones of Regulation Module	
8. Caregiver selected self-regulation as intervention priority 9. Verbally fluent as defined by ADOS-2 module 3 criteria	
Additional Inclusion Criteria for Equine Environment	
10. Meet PATH, Intl medical and behavioral standards, including obtaining a physician signature 11. Can ride a horse for 10 minutes while following safety rules	

ASD, Autism Spectrum Disorder; ADOS-2, Autism Diagnostic Observation Schedule, Second Edition; ABC-C, Aberrant Behavior Checklist, Community; CASI-5, Child and Adolescent Symptom Inventory, Fifth Edition; PATH Intl, Professional Association of Therapeutic Horsemanship, International.

### 2.3 Pre-intervention initial visit

The first study visit involved an occupational therapy evaluation and goal setting with an occupational therapist. The occupational therapy evaluation consisted of a) an occupational profile addressing youth’s self-regulation, play, social participation, and education, and b) a 10-item self-regulation skill checklist with skills related to emotional awareness and use of self-regulation strategies. Next, the caregiver and therapist collaboratively set three individual goals related to three different self-regulation areas: understanding emotions, identifying self-regulation tools, and using self-regulation tools. Youth were invited to participate in this collaborative goal setting to the extent that they were able, unless the caregiver specifically requested not to involve youth in goal-setting due to self-esteem. After the first visit, the occupational therapist scaled goals using goal-attainment scaling methods described below.

### 2.4 OT-ZOR clinic intervention

Peters et al. (28) provides a thorough description of the OT-ZOR Clinic intervention, summarized here. OT-ZOR occurred in a private clinic playroom that included several swings, a rock-climbing wall, individual trampoline, scooter boards, a table and chairs, social turn-taking games, and general therapy supplies such as cones, beanbags, and bolsters. Two licensed and registered occupational therapists provided the OT-ZOR Clinic intervention. One occupational therapist had been licensed for three years, had experience providing occupational therapy to autistic youth, and was previously familiar with the Zones of Regulation curriculum. The other occupational therapist had been licensed for six years, had experience providing occupational therapy to autistic youth, but had limited previous exposure to the Zones of Regulation curriculum. Each therapist received 10 hours of training specific to the OT-ZOR Clinic manual and participated in five one-hour case conferences throughout both 10-week intervention sessions. Case conferences focused on ensuring fidelity to the intervention manual and identifying methods to address participant-related challenges.

The first author paired autistic youth into dyads after the initial pre-intervention visit based on social communication and self-regulation abilities. Each of these two participants was assigned their own occupational therapist, attempting to pair children with the same occupational therapist who performed the pre-intervention visit when scheduling allowed. Paired youth received therapy in the same space at the same time, which allowed youth to practice emerging skills with a social partner. Dyads attended ten weekly 60-minute sessions of occupational therapy in a clinic that followed a general structure: greeting, activities in a playroom, parent debrief, and goodbyes. The OT-ZOR Clinic manual includes 10 activities adapted from the original Zones of Regulation™ (36) curriculum, each activity addressing one of the following self-regulation skills:

- Categorizing emotions and alertness states into four Zones
- Recognizing emotions in facial expressions
- Identifying expected Zones for different situations
- Identifying body cues for different Zones
- Identifying individualized triggers
- Identifying sensory regulation tools
- Identifying calming regulation tools
- Identifying cognitive regulation tools
- Creating an individualized regulation toolbox, and
- Using an individualized regulation toolbox

These original Zones of Regulation™ activities were modified for an outpatient occupational therapy context and to fulfill critical elements of OT-ZOR Clinic in **Table 2**. For example, to work on the first skill of categorizing emotions and alertness states into four zones, the occupational therapists taught youth about the blue, green, yellow, and red zones (**Table 2** element #2- direct instruction). Next youth played a game by pulling a card with an emotion on it and acting out the emotion; the other child guessed the emotion and corresponding zone (element #3- therapeutic play activity). Youth then rode scooters to a poster that corresponded with the zone color and taped the emotion onto the poster (element 4- positive reinforcement using preferred activity). Throughout the activity the occupational therapists integrated children’s strengths and interests (element #1), and scaffolded their ability to categorize them emotion into a zone (element #5). While joint activities were planned for every session (barring an absence), the level of engagement between youth varied based on youth’s social abilities and social interest.

**Table 2:** Critical intervention elements.

OT-ZOR Equine	OT-ZOR Clinic
1. Support attention and engagement through use of a) equine movement (i.e., hippotherapy) to facilitate optimal physiological arousal, b) strengths & interests, including preferred equine activities, and c) regulation tools.	1. Support attention and engagement through use of strengths, interests, and regulation tools
2. Provide direct instruction of a self-regulation skill from Zones of Regulation™	2. Provide direct instruction of a self-regulation skill from Zones of Regulation™
3. Offer therapeutic activities with horses to practice the self-regulation skill	3. Offer therapeutic play activities to practice the self-regulation skill
4. Give positive reinforcement for practicing the weekly self-regulation skill (preferred equine activities often the reinforcement)	4. Give positive reinforcement for practicing the weekly self-regulation skill
5. Scaffold skill performance using prompting, fading, shaping, chaining, and feedback	5. Scaffold skill performance using prompting, fading, shaping, chaining, and feedback
6. Create an environment to best support skill performance (horse selection, tack selection, arena set-up)	6. Create an environment to best support individual performance

### 2.5 Mid-intervention visit

After 10 weeks of OT-ZOR Clinic, youth attended an additional visit (**Figure 2**) to ensure they met additional inclusion criteria for the equine environment listed in **Table 1**. This screening portion included riding a horse for 15 minutes while following safety rules. Then, caregivers and youth collaborated with the occupational therapist to update or set new goals for 10 weeks of OT-ZOR Equine.

### 2.6 OT-ZOR Equine intervention

The OT-ZOR Equine intervention occurred in a large indoor arena meant specifically for equine-assisted services, with available toys such as beanbags, cones, balls, a ring “tree” and rings, basketball hoop, mailbox, and poles. The intervention was provided by two licensed and registered occupational therapists. One occupational therapist had been licensed for 15 years and the other for 6 years. Both had Level 2 training from the American Hippotherapy Association, were also PATH Intl registered instructors, and had experience delivering occupational therapy in an equine environment to autistic youth. One therapist had prior experience using the Zones of Regulation curriculum, while the other had only limited prior exposure to the curriculum. These therapists were different therapists than those who provided the OT-ZOR Clinic intervention. Therapists received 10 hours of training specific to the OT-ZOR Equine manual and participated in five one-hour case conferences throughout both 10-week intervention sessions.

The same dyads from the 10 weeks of OT-ZOR Clinic were maintained. Dyads attended 10 weekly 60-minute sessions of OT-ZOR Equine. Sessions followed a general structure: greetings, activities with horses, caregiver debrief, goodbyes. The same 10 self-regulation skills from the Zones of Regulation™ curriculum were adapted to be delivered in an equine environment and to fulfill the critical elements in **Table 2**, which intentionally mirrored all critical elements of OT-ZOR Clinic, but with the addition of purposeful inclusion of horses to augment the intervention (in critical elements #1, #3, #4, and #6).

For example, to work on the first skill of categorizing emotions and alertness states into four zones, the occupational therapists provided the same direct instruction and emotion charades games as previously described for OT-ZOR Clinic, while participants were mounted on a horse (**Table 2** elements #2 and #3). Youth then chose how to ride their horse (e.g., walk, trot, side-sitting, etc.) to a barrel with a poster that corresponded with the zone color and taped the emotion onto the poster (element #4- positive reinforcement using preferred activity). Throughout the activity the occupational therapists integrated youths’ strengths and interests, including fostering their interest in the horse (element #1), and scaffolded their ability to categorize the emotion into a zone (element #5). Also like OT-ZOR Clinic, joint activities were planned for every session (barring an absence), but the level of engagement between youth varied based on youth’s social abilities and social interest.

### 2.7 Data collection

#### 2.7.1 Aim 1: participant recruitment, attendance, and retention

**Table 3** includes benchmarks for each feasibility indicator. We used Excel spreadsheets to monitor recruitment, reasons for ineligibility, attendance, withdrawals, and reasons for withdrawals.

**Table 3:** Feasibility indicators for intervention implementation and acceptability.

Feasibility Indicator	Feasibility Benchmark	Result
Participant Recruitment	20 participants	20 participants (this paper reports on 14 assigned to OT-ZOR)
Participant Retention	90%	100%
Participant Attendance	90%	95%
Intervention Fidelity	90%	91%
Assessment Completion	90%	93% ABC-C, EDI, SRS-2, WHOQOL- Bref; 50% PEDICAT-ASD
Adverse Events	0 serious adverse events	0 serious adverse events
Caregiver Acceptability	90% satisfied or very satisfied	100% satisfied or very satisfied
Occupational Therapist Acceptability	90% satisfied or very satisfied	100% satisfied or very satisfied

ZOR, Zones of Regulation^TM^; ABC-C, Aberrant Behavior Checklist-Community; EDI, Emotional Dysregulation Inventory; SRS-2, Social Responsiveness Scale, Second Edition; WHOQOL-Bref, World Health Organization Quality of Life Assessment, Brief Version.

#### 2.7.2 Aim 2: OT-ZOR Equine intervention fidelity, safety & assessment completion

We monitored intervention fidelity using the OT-ZOR Equine fidelity rating form created for this study. The rating form measures the presence and quality of the structural and critical elements of OT-ZOR Equine described above. The first author and a graduate research assistant obtained 97% agreement on use of the OT-ZOR Equine fidelity rating form by jointly rating 8 sessions. The first author then rated the fidelity of 26% of OT-ZOR Equine sessions.

We monitored the safety of OT-ZOR Equine using incident report forms completed by occupational therapists that detailed any adverse events that occurred during the intervention.

#### 2.7.3 Aim 3: therapist and caregiver acceptability of OT-ZOR Equine

After OT-ZOR Equine, caregivers completed a satisfaction survey with Likert-scale questions that addressed: perceived benefits of OT-ZOR Equine, the intervention’s goodness-of-fit, logistics of attending, and youth’s perceived enjoyment/willingness to attend. Open-ended questions asked caregivers about the best and worst aspects of OT-ZOR Equine, and suggestions for improvement.

Both occupational therapists completed an online satisfaction survey after delivering two 10-week sessions of OT-ZOR Equine. Likert-scale and open-ended questions focused on overall satisfaction, perceived appropriateness of OT-ZOR Equine, logistical feasibility, intent to continue use, and suggestions for improvement.

Both occupational therapists also participated in a focus group online through Microsoft Teams after two 10-week sessions of OT-ZOR Equine. The first author led the focus group using a semi-structured guide that included open-ended questions about overall satisfaction, perceived appropriateness of OT-ZOR Equine, logistical feasibility, intent to continue use, and suggestions for improvement.

#### 2.7.4 Aim 4: preliminary participant outcomes

Caregivers completed outcomes measures online three times: Time 1 pretest, Time 2 after OT-ZOR Clinic, and Time 3 after OT-ZOR Equine. All caregiver-completed outcome assessments were completed on a secure web-based platform called REDCap, with the exception of one assessment that had to be completed on Pearson’s secure online Q-Global platform (the Pediatric Evaluation of Disability Inventory Computer Adaptive Test).

##### 2.7.4.1 Aberrant Behavior Checklist, Community (ABC-C)

Caregivers completed the irritability and hyperactivity scales of the ABC-C (37), a measure of the presence and severity of problem behaviors. In the absence of targeted interventions, test-retest reliability of caregiver-ratings on the ABC-C is stable, ranging from r=0.80 to r=0.95. This measure, frequently used in clinical trials in ASD research (38), has concurrent validity with other measures of behavior (37).

##### 2.7.4.2 Emotional Dysregulation Inventory (EDI)

The EDI (39) is a 30-item assessment that quantifies emotional dysregulation in autistic youth. The EDI, which was developed with methods from item response theory, results in a total score for emotional reactivity (“intense, rapidly escalating, sustained, and poorly regulated negative emotional reactions” p. 928) and dysphoria (“characterized by anhedonia, sadness, and nervousness” p. 928). It has concurrent validity with other behavioral measures of emotional dysregulation, anxiety, depression, irritability, hyperactivity, and aggression; has good test-retest reliability in the absence of interventions (emotional reactivity mean difference = 0.05; dysphoria mean difference = 0.02); and is sensitive to changes in emotional dysregulation following intervention (emotional reactivity mean difference = 1.21; dysphoria = 0.70).

##### 2.7.4.3 Social Responsiveness Scale, Second Edition (SRS-2)

The SRS-2 (40) is a 65-item questionnaire that measures autism-specific social functioning in five subscales: social awareness, social cognition, social motivation, social communication, and restricted interests and repetitive behaviors. The SRS-2 has evidence of internal consistency, Cronbach’s α=0.95 for clinical samples, test-retest reliability ranging between r=0.88 and r=0.95, and concurrent validity with other social behavior measures (41).

##### 2.7.4.4 World Health Organization Quality of Life- BREF (WHOQOL-BREF)

The WHOQOL-BREF (42) is a 26-item measure of caregiver quality-of-life across four domains: physical health, psychological, social relationships, and environment. This measure has established discriminant and content validity, good test-retest reliability (Cronbach’s α=0.66 – 0.84), and has been validated in parents of autistic youth (43).

##### 2.7.4.5 Pediatric Evaluation of Disability Inventory Computer Adaptive Test for Autism Spectrum Disorder (PEDICAT-ASD)

The PEDICAT-ASD (44) is a caregiver-report measure of child performance and participation in 4 functional areas: mobility, social/cognitive, daily activities, and responsibility. This version of the PEDICAT was modified specifically for autistic youth, with excellent test-retest reliability in the absence of intervention (ICC ≥0.86) and concurrent validity with other measures of adaptive behavior.

##### 2.7.4.6 Goal Attainment Scaling (GAS)

Goal attainment scaling is a standardized method for measuring progress on individual, functional goals and is regarded as a useful outcome measure of individual progress in intervention studies for ASD (45). The first author trained occupational therapists in implementation of GAS according to procedures described by McDougall and King (46). Occupational therapists collaboratively determined goal areas with parents and participants via a standardized semi-structured interview, in line with current GAS recommendations (45, 47). Guided by this semi-structured interview, self-regulation goals pertained to 1) identifying emotions, 2) identifying self-regulation tools/strategies, and 3) using self-regulation tools/strategies. Occupational therapists used a template to ensure that goals were systematically scaled across participants. Per this method, occupational therapists scaled goals onto a 5-point scale, where -2 indicated the level of performance at the time of evaluation, -1 indicated less-than-expected level of performance after the intervention, 0 indicated the expected level of performance after the intervention (the “goal level”), +1 indicated more-than-expected performance after the intervention, and +2 indicated much-more-than-expected performance after the intervention. The first author verified that all defined goals both addressed self-regulation skills and met the criteria outlined in McDougall and King’s (46) GAS checklist.

Following each ten-week session of OT-ZOR Clinic or OT-ZOR Equine, an occupational therapist blinded to the treatment conditions of each participant conducted a semi-structured interview with each participant’s caregiver in order to rate the youth’s goal attainment on their individual goals. Parents remained blinded to the numerical values of each rating, as well as to the specific behavioral benchmarks in the GAS scales. As participant behavior tends to improve in the context of therapy, we chose to depend upon caregiver-report of participants’ performance to ensure GAS ratings were representative of participant behavior in home and community contexts. In order to establish interrater reliability, AH listened to a random 69% of recorded caregiver interviews and rated the child’s goal attainment.

### 2.8 Data analysis

We used Microsoft Excel to calculate descriptive statistics of quantitative feasibility and acceptability data. Focus group data was transcribed verbatim using Microsoft Word and uploaded into NVivo Qualitative Software. Open-ended survey responses were also uploaded into NVivo. AH conducted a directed content analyses (48) of qualitative data using Nvivo Qualitative software. Content analyses began with pre-determined parent codes derived from indicators of acceptability (49). AH then inductively generated child codes within each parent code, and new parent codes as needed. Next, she created narrative summaries of the data, informed by peer-review from the first author.

To assess preliminary participant outcomes, we conducted a repeated measures ANOVA using a linear mixed effects model with unstructured covariance to compare outcomes at three time-points: Time 1 pretest, Time 2 after OT-ZOR Clinic, and Time 3 after OT-ZOR Equine. For the analysis, we included participants with outcome data at 2 or more timepoints: Time 1 & 2 (n=1) and Time 1, 2, and 3 (n=13). No adjustment for type I errors for multiple comparisons or multiple outcomes were applied for this exploratory feasibility study. P-value < 0.05 was deemed statistically significant. All p-values are reported.

## 3 Results

**Figure 2** is a modified CONSORT diagram that illustrates participant progression through the study. **Table 4** presents demographic and clinical characteristics of participants who completed OT-ZOR Clinic and OT-ZOR Equine, and their caregivers. **Table 3** presents results of each feasibility indicator, as next described.

**Table 4:** Participant and caregiver characteristics.

Participant Characteristic	Mean ± SD or Count N=14
Age	8.8 ± 2.6
ABAS-3 General Adaptive Composite	74.5 ± 11.88
SCQ Total	20.0 ± 4.3
Total Irritability Score	24.3 ± 7.8
Sex (M/F)	6/8
Race	
Black or African American	1
White	10
Multiracial	3
Ethnicity (Hispanic or Latino)	0
Household Income	
≤ $50,000	6
$51,000 - $100,000	4
>100,000	4
Caregiver Age	35.7 ± 4.8
Caregiver Sex (M/F)	1/13
Caregiver Race	
White	13
American Indian/Alaskan Native	1
Caregiver Ethnicity (Hispanic or Latino)	0

ABAS-3, Adaptive Behavior Assessment System, Third Edition; SCQ, Social Communication Questionnaire.

### 3.1 Aim 1: participant recruitment, attendance, and retention

Twenty-six participants were assessed for eligibility, six were excluded because they did not meet the ABC-C irritability inclusion criteria (n=4) or they chose not to participate (n=2). Ten participants were enrolled into the first cohort within three months of recruitment, and an additional ten were enrolled in the second cohort within an additional four months. Recruitment ended after 20 participants enrolled in the study. Fourteen of 20 participants met inclusion criteria for OT-ZOR Equine (**Table 1**) and are therefore included in the results of this paper. No participants discontinued the OT-ZOR Equine intervention, resulting in 100% retention. In line with exclusion criteria three (**Table 1**), we monitored participant therapy changes throughout the study period; no youth started new therapies during the study period. Youth attended 95% of OT-ZOR Equine sessions; seven participants missed one session each.

### 3.2 Aim 2: OT-ZOR Equine intervention fidelity, safety, and assessment completion

On average, both occupational therapists attained 91% fidelity to the OT-ZOR Equine intervention; fidelity scores ranged from 72% to 100%.

No serious adverse events occurred during OT-ZOR Equine. One non-serious adverse event occurred; a horse briefly nipped a participant on the arm, causing mild bruising. No follow-up medical care was required, and no other safety events were reported throughout the OT-ZOR Equine intervention.

Thirteen caregivers completed five out of six outcome assessments after OT-ZOR Equine; one caregiver was lost to follow-up as she no longer lived in the same household as her child at the post-test assessment. However, only seven caregivers completed the PEDICAT-ASD outcome measure, likely because it had to be completed on a different online platform (Pearson’s Q-Global) than the rest of the outcome battery.

### 3.3 Aim 3: OT-ZOR Equine acceptability

#### 3.3.1 Caregiver acceptability survey

One hundred percent of caregivers who completed the acceptability survey (n=13) agreed or strongly agreed they were overall satisfied with the OT-ZOR Equine intervention. Caregivers were most satisfied with the goals the intervention addressed, the rapport their child built with the occupational therapist, and reported they would recommend the OT-ZOR Equine intervention to another caregiver with an autistic child (100% agreed or strongly agreed). Ninety-two percent of caregivers agreed or strongly agreed that OT-ZOR Equine was a good fit for their child, and that their child enjoyed it. Eighty-five percent of caregivers believed OT-ZOR Equine was beneficial for their child, and that their child was agreeable to attend. However, only 62% of caregivers agreed or strongly agreed that OT-ZOR Equine was logistically feasible to attend (time of day, driving distance, etc.).

In the open-ended responses, caregivers expressed overall satisfaction with the OT-ZOR Equine intervention, particularly that their children not only *“loved”* their experiences in OT-ZOR Equine, but also demonstrated observable improvements following the intervention. Caregivers expressed satisfaction with their children’s “*opportunity to work with horses*” throughout the OT-ZOR intervention, one caregiver stating, *“We found out he absolutely loves horses and the riding had such a calming effect on him.”* Caregivers were complimentary of the high-quality, trusting therapist-client relationships their children formed with the occupational therapists, as well as of the “*new skills*” that their children learned via OT-ZOR Equine. There were few negative comments regarding the intervention, with a few critiques concerning the long commute to the facility on the outskirts of town and that OT-ZOR Equine sessions were often scheduled during school hours (due to the facility’s 8am-5pm schedule). Overall, parents had few suggestions to improve the OT-ZOR Equine intervention, with some parents noting that they would like the option for more after-school scheduling availability as well as a greater frequency of sessions throughout the study.

#### 3.3.2 Occupational therapist acceptability survey

Both occupational therapists agreed or strongly agreed that they were satisfied with the OT-ZOR Equine intervention, particularly that it was appropriate for autistic youth and logistically feasible to implement. They both agreed or strongly agreed that they would continue to implement the OT-ZOR Equine intervention after the study concluded, and that they would recommend OT-ZOR Equine to other occupational therapists.

In response to open-ended survey questions, occupational therapists regarded the structure of the intervention and the presence of the horses as the salient positive aspects of OT-ZOR Equine intervention, stating, *“participants seemed responsive and motivated using equines,”* and, *“The equines allowed for pressure to be taken off the youth and anxiety decreased when practicing [self-regulation skills]”* When asked about the worst part of OT-ZOR Equine, occupational therapists referenced the time commitment required to complete the documentation forms.

#### 3.3.3 Occupational therapist focus group

Overall, occupational therapists were satisfied with their experiences taking part in this study and noted particular satisfaction with the content of the OT-ZOR Equine evaluation and intervention, the goal attainment scaling process, and the OT-ZOR Equine manual. Therapists expressed intent to incorporate several components of this study’s evaluation process into their clinical practice, including goal attainment scaling and use of the occupational profile. Therapists suggested that teenage participants may benefit from more detailed descriptions about OT-ZOR Equine prior to the start of their participation: “*That would have been helpful, so that he knew what we were going to talk about.*” Occupational therapists considered OT-ZOR Equine to be occupation-based, and appropriate for occupational therapists to deliver to autistic youth in an equine environment. They noted that multiple participants were motivated by unmounted equine activities, such as groundwork, and suggested the addition of unmounted activities in future studies, even if that addition would slightly decrease mounted time. As well, while occupational therapists generally reacted positively to the Zones of Regulation™ curriculum, they felt that increased training time on the program prior to implementation, and a more structured implementation scheme—such as introducing one Zones of Regulation™ *tool* to participants at the start of each intervention session—would both help the therapists better understand the program and expose youth participants to the curriculum’s tools more naturally.

### 3.4 Aim 4: OT-ZOR Equine preliminary participant outcomes

**Table 5** presents preliminary participant outcomes.

**Table 5:** Participant outcomes.

Outcome	*F*	Comparison	Difference	DF	*t*	*p*
**ABC-C Irritability**	8.74**	**(1. Baseline) - (2. Clinic)**	**5.6**	**13**	**2.79**	0.015*
		**(1. Baseline) - (3. Equine)**	**6.9**	**13**	**4.16**	0.001**
		(2. Clinic) - (3. Equine)	1.3	13	0.96	0.356
**ABC-C Hyperactivity**	7.57**	**(1. Baseline) - (2. Clinic)**	**5.8**	**13**	**2.80**	0.015*
		**(1. Baseline) - (3. Equine)**	**7.1**	**13**	**3.89**	0.002**
		(2. Clinic) - (3. Equine)	1.3	13	0.94	0.365
**EDI Emotional Reactivity**	7.74**	**(1. Baseline) - (2. Clinic)**	**4.4**	**13**	**2.61**	0.021*
		**(1. Baseline) - (3. Equine)**	**5.1**	**13**	**3.85**	0.002**
		(2. Clinic) - (3. Equine)	0.7	13	0.44	0.664
**EDI Dysphoria**	6.78*	**(1. Baseline) - (2. Clinic)**	**3.8**	**13**	**2.80**	0.015*
		**(1. Baseline) - (3. Equine)**	**5.1**	**13**	**3.61**	0.003**
		(2. Clinic) - (3. Equine)	1.3	13	1.08	0.301
SRS-2 Social Awareness	0.71	(1. Baseline) - (2. Clinic)	-1.7	13	-0.81	0.431
		(1. Baseline) - (3. Equine)	0.7	13	0.39	0.699
		(2. Clinic) - (3. Equine)	2.4	13	1.19	0.256
**SRS-2 Social Cognition**	4.52*	(1. Baseline) - (2. Clinic)	1.6	13	0.54	0.596
		**(1. Baseline) - (3. Equine)**	**4.0**	**13**	**2.24**	0.043*
		(2. Clinic) - (3. Equine)	2.4	13	1.32	0.209
**SRS-2 Social Communication**	9.90**	(1. Baseline) - (2. Clinic)	-1.1	13	-0.62	0.547
		**(1. Baseline) - (3. Equine)**	**4.0**	**13**	**2.21**	0.045*
		**(2. Clinic) - (3. Equine)**	**5.1**	**13**	**4.42**	<.001**
SRS-2 Social Motivation	1.25	(1. Baseline) - (2. Clinic)	3.2	13	1.53	0.149
		(1. Baseline) - (3. Equine)	1.2	13	0.54	0.601
		(2. Clinic) - (3. Equine)	-2.0	13	-0.97	0.350
SRS-2 RRB	2.00	(1. Baseline) - (2. Clinic)	1.6	13	0.83	0.423
		(1. Baseline) - (3. Equine)	4.4	13	1.93	0.075
		(2. Clinic) - (3. Equine)	2.8	13	1.50	0.158
SRS-2 Total	5.40*	(1. Baseline) - (2. Clinic)	0.4	13	0.21	0.833
		**(1. Baseline) - (3. Equine)**	**3.8**	**13**	**2.20**	0.046*
		**(2. Clinic) - (3. Equine)**	**3.4**	**13**	**2.72**	0.017*
**WHOQOL-BREF Physical Health**	4.05*	(1. Baseline) - (2. Clinic)	0.0	13	0.00	1.000
		**(1. Baseline) - (3. Equine)**	**-1.2**	**13**	**-2.35**	0.035*
		**(2. Clinic) - (3. Equine)**	**-1.2**	**13**	**-2.49**	0.027*
WHOQOL- BREF Psychological	2.10	(1. Baseline) - (2. Clinic)	-0.7	13	-1.73	0.106
		(1. Baseline) - (3. Equine)	-0.6	13	-0.85	0.409
		(2. Clinic) - (3. Equine)	0.2	13	0.42	0.681
WHOQOL-BREF Social Relationships	1.9	(1. Baseline) - (2. Clinic)	-1.5	13	-1.93	0.075
		(1. Baseline) - (3. Equine)	-1.1	13	-1.27	0.228
		(2. Clinic) - (3. Equine)	0.4	13	0.53	0.602
WHOQOL-BREF Environment	0.35	(1. Baseline) - (2. Clinic)	-0.4	13	-0.69	0.500
		(1. Baseline) - (3. Equine)	-0.4	13	-0.67	0.512
		(2. Clinic) - (3. Equine)	0.1	13	0.12	0.907

ABC-C, Aberrant Behavior Checklist-Community; EDI, Emotional Dysregulation Inventory; SRS-2, Social Responsiveness Scale, Second Edition; WHOQOL-Bref, World Health Organization Quality of Life Assessment, Brief Version; Clinic, Occupational Therapy using Zones of Regulation™ Concepts in a Clinic; Equine, Occupational Therapy using Zones of Regulation™ Concepts in an Equine Environment.

Bold indicates p<0.05.

* indicates p<0.05; ** indicates p<0.01.

#### 3.4.1 OT-ZOR clinic fidelity & attendance

While Peters et al. (28) provides a full report on the feasibility of OT-ZOR Clinic, to provide context for the analyses of preliminary participant outcomes below, in this paper we report attendance and fidelity. Participants attended 94% of OT-ZOR Clinic sessions, with eight participants missing one session each. Providers achieved 97% fidelity to the OT-ZOR Clinic intervention.

#### 3.4.2 Self-regulation

**Figure 3:**
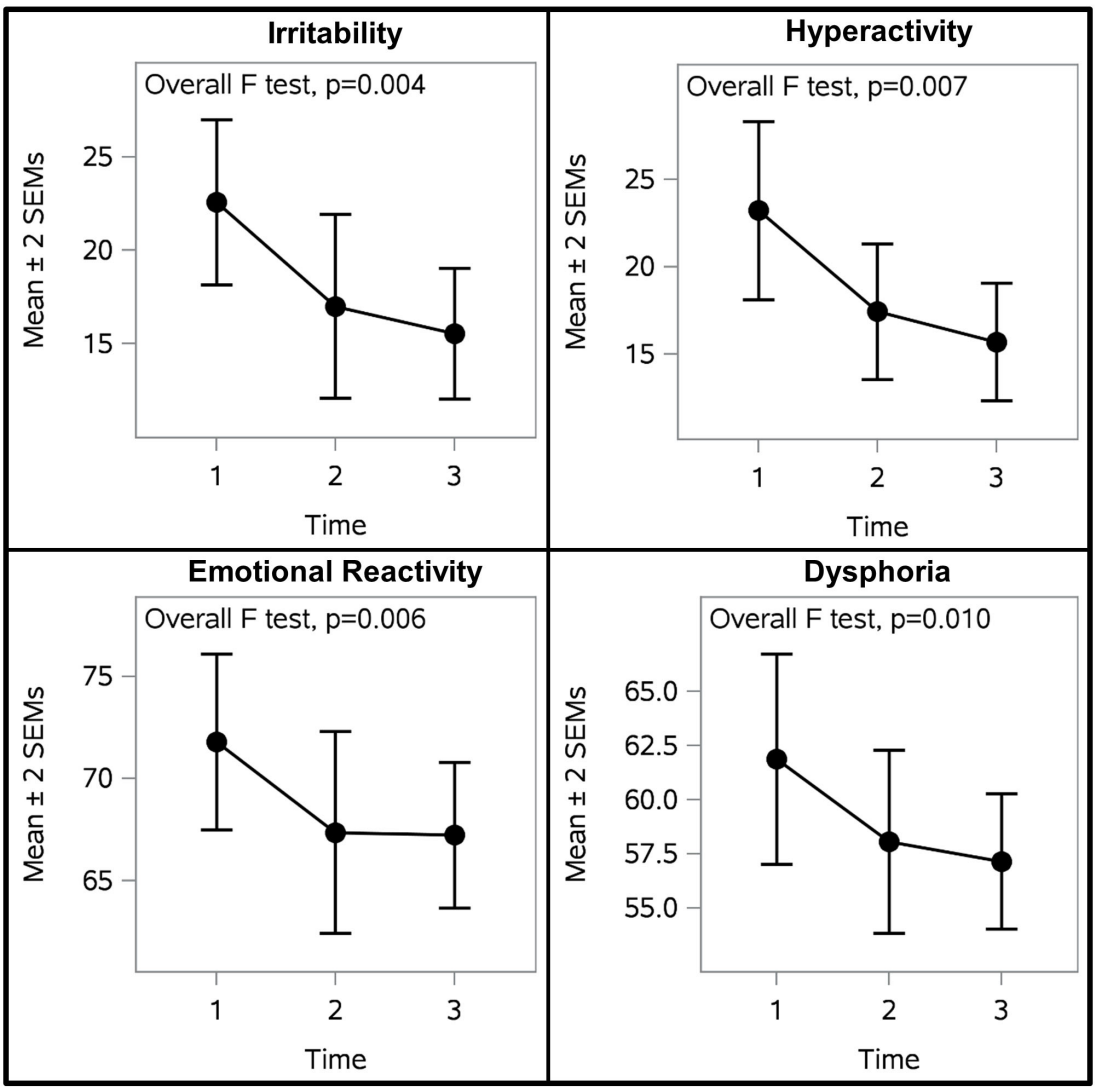
Participant Self-regulation Outcomes. Participants demonstrated significnatly improved self-regulation across all four self-regulation indicators. The largest improvements occurred after OT-ZOR Clinic (Time 2). Improvements were maintained but plateaued after OT-ZOR Equine (Time 3). Time 1=Baseline; Time 2= After OT-ZOR Clinic; Time 3=After OT-ZOR Equine.

**Figure 3** illustrates participant outcomes on indicators of self-regulation as measured by the ABC-C and EDI. There were statistically significant differences between measurement timepoints in irritability, hyperactivity, emotional reactivity, and dysphoria (**Table 5**). Mixed model analyses revealed that each of these indicators of self-regulation were significantly improved after OT-ZOR Clinic (Time 2) and OT-ZOR Equine (Time 3) in comparison to baseline (Time 1). There were no significant differences in any of these self-regulation indicators when comparing post-OT-ZOR Clinic (Time 2) and post-OT-ZOR Equine (Time 3). Overall then, participants demonstrated the greatest improvements in self-regulation after OT-ZOR Clinic, and these improvements maintained but plateaued after OT-ZOR Equine. It is worth noting, after 10 weeks of OT-ZOR Clinic several participants had dropped below the inclusion criteria of ABC-C irritability scores >10, therefore, the plateau in improvement in self-regulation indicators after OT-ZOR Equine could represent a floor effect.

#### 3.4.3 Social functioning

**Figure 4:**
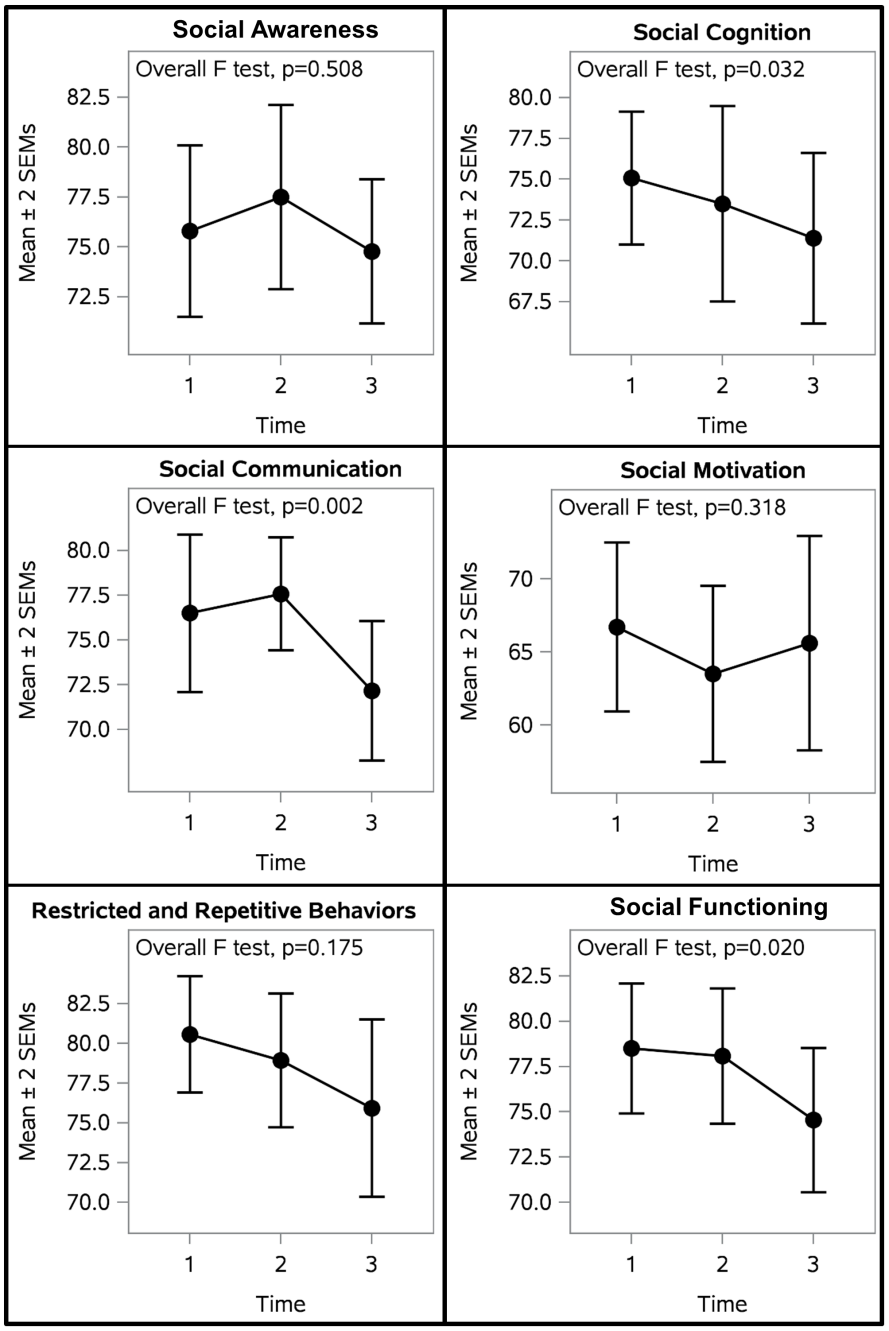
Participant Social Outcomes. Participants demonstrated significantly improved social cognition, social communication, and total social functioning. There were no significant differences after OT-ZOR Clinic (Time 2), but significant improvements after OT-ZOR Equine (Time 3). Time 1=Baseline; Time 2= After OT-ZOR Clinic; Time 3=After OT-ZOR Equine.

**Figure 4** illustrates participant outcomes on indicators of social functioning, as measured by the SRS-2. There were significant differences between measurement timepoints in total social functioning, social communication, and social cognition (**Table 5**). Mixed model analyses revealed that total social functioning and social communication were significantly improved after OT-ZOR Equine (Time 3) in comparison to baseline and post-OT-ZOR Clinic (Times 1&2). Furthermore, social cognition was significantly improved after OT-ZOR Equine (Time 3) in comparison to baseline (Time 1). There were no significant differences in any of these indicators of social functioning after OT-ZOR Clinic (Time 2) compared to baseline (Time 1). Overall then, participants did not demonstrate significant improvements in social functioning after OT-ZOR Clinic, but then demonstrated significant improvements particularly in social communication and social cognition after OT-ZOR Equine.

#### 3.4.4 Caregiver quality of life

There were significant differences between measurement timepoints in the physical health domain of caregiver quality of life of the WHOQOL-Brief (**Table 5**). Mixed model analyses revealed caregivers’ physical health was greater after OT-ZOR Equine (Time 3) in comparison to baseline or post-OT-ZOR Clinic (Times 1 & 2). There were no significant differences in caregivers’ physical health after OT-ZOR Clinic (Time 2) in comparison to baseline (Time 1). Overall, caregivers’ quality of life did not change after OT-ZOR Clinic, but was significantly improved in the domain of physical health after their child participated in the OT-ZOR Equine intervention.

#### 3.4.5 Goal attainment

**Figure 5:**
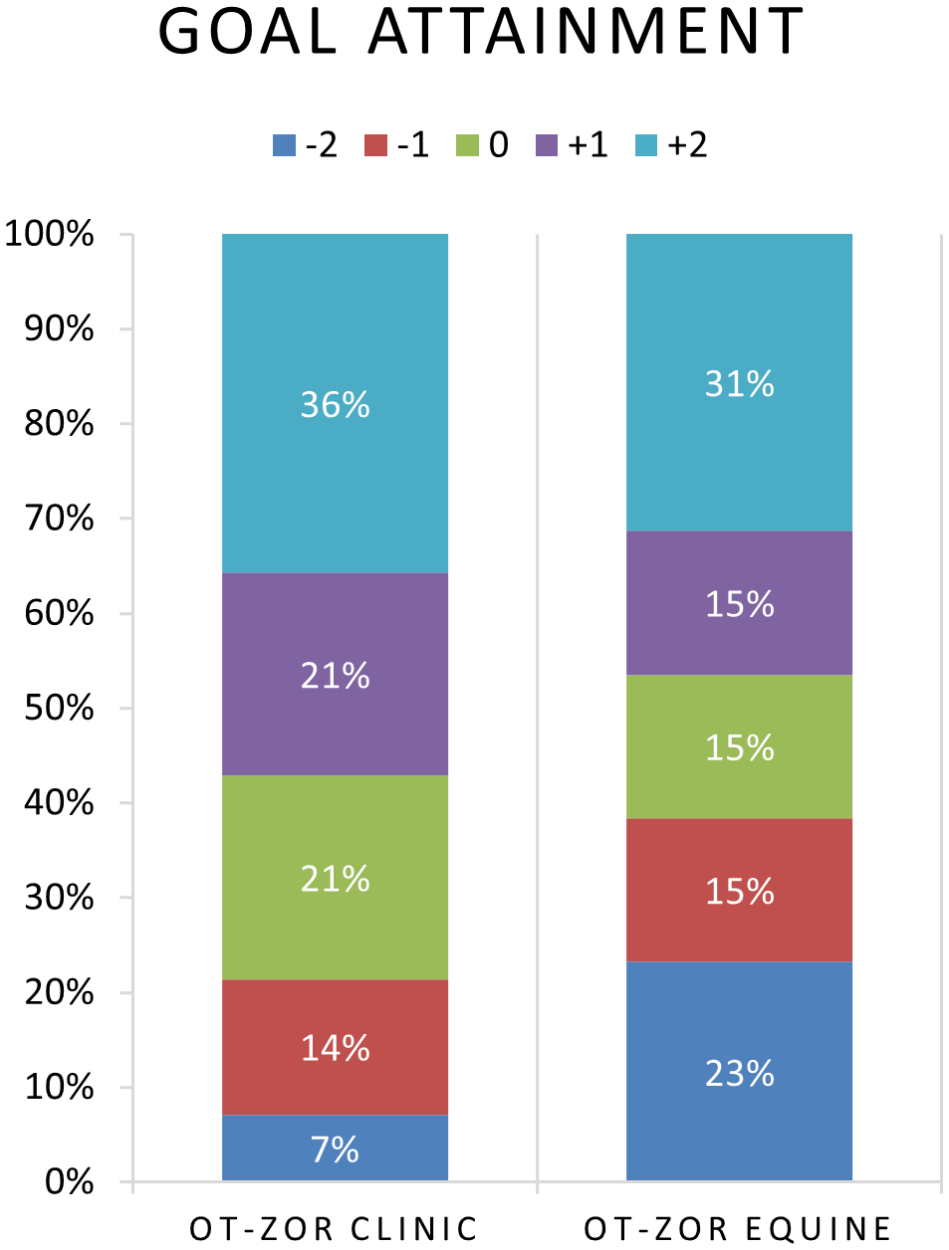
Distribution of goal attainment ratings after OT-ZOR Clinic and OT-ZOR Equine. OT-ZOR Clinic=Occupational Therapy using Zones of Regulation™ Concepts in a Clinic; OT-ZOR Equine=Occupational Therapy using Zones of Regulation™ Concepts in an Equine Environment. -2=level of performance at the time of evaluation; -1= less-than-expected level of performance after the intervention; 0=expected level of performance after the intervention; +1=more-than-expected performance after the intervention; +2=much-more-than-expected performance after the intervention.

**Table 6** provides example participant goals. **Figure 5** illustrates participant progress on individual self-regulation goals after OT-ZOR Clinic and OT-ZOR Equine. We attained 94% interrater reliability on goal ratings. Seventy-nine percent of participants met or exceeded their individual occupational performance goal related to self-regulation after participation in OT-ZOR Clinic, as indicated by a post-test GAS score of 0, + 1, or +2. Sixty-one percent of participants met or exceeded their primary individual occupational performance goal related to self-regulation after participation in OT-ZOR Equine, as indicated by a post-test GAS score of 0, + 1, or +2.

**Table 6:** Example self-regulation goals.

Self-regulation Area	Example Goal
Understanding Emotions	Jaime names a complex emotion in himself when becoming dysregulated (e.g., disappointed, overwhelmed) with 2 verbal cues in 80% of opportunities.
Identifying self-regulation tools	Emma names a self-regulation tool that can help her calm down when provided with a verbal or visual prompt by her parent.
Using self-regulation tools	Micah uses a self-regulation tool while in the “yellow zone” (starting to become upset) 70% of the time with 3 verbal or visual cues from mom.

## 4 Discussion

This paper aimed to evaluate the feasibility of an updated intervention protocol, OT-ZOR Equine, specifically evaluating: 1) participant recruitment, retention, and attendance, 2) intervention fidelity, safety, and caregiver assessment completion, 3) acceptability of the OT-ZOR Equine intervention to providing occupational therapists and caregivers of autistic youth, and 4) preliminary participant outcomes after the OT-ZOR Equine intervention.

We found the intervention and study protocol largely feasible to implement, as demonstrated by exceeding feasibility benchmarks in recruitment, retention, attendance, fidelity, and safety. We successfully recruited 20 participants within a reasonable timeframe, notably recruiting more female than male participants. Historically females are underrepresented in autism research (50–52), our success in recruiting female participants may be attributed to the inclusion of horses in our recruitment materials, as autistic girls identify animals as a special interest more often than autistic boys (53, 54). However, the sample of participants in the current study was not racially or ethnically diverse; recruitment efforts in future studies should partner with local communities to intentionally recruit a more diverse sample.

Assessing intervention acceptability is a critical research task in designing and evaluating interventions (29, 55), as an intervention’s acceptability to both the recipient and the healthcare provider may affect its implementation, participant adherence, and overall effectiveness (56). Caregivers generally found the OT-ZOR Equine intervention acceptable, as evidenced by 100% satisfaction rates, 100% retention in the study, and high attendance. This mirrors the high acceptability ratings of caregivers of youth who participated in the OT^ee^ HORSPLAY intervention (27), suggesting the modifications to the OT-ZOR Equine intervention protocol to increase standardization did not decrease caregiver acceptance. Following advice from caregiver’s suggested improvements, future studies can offer more after-school times and offer OT-ZOR Equine at facilities closer to suburbs/cities (as opposed to the more rural location in the current study). Furthermore, the caregiver outcome battery was largely feasible, apart from one assessment that had to be completed on a different online platform (PEDICAT-ASD). Future OT-ZOR Equine research should use an outcome measure of adaptive behavior that can be completed on the same platform as all other outcome measures, and could recruit teachers to report on participants’ behavior at school, to assess if potential improvements in social functioning and self-regulation have an impact on youths’ daily lives.

Similarly, occupational therapists found the OT-ZOR Equine intervention acceptable, and they were able to implement it safely and with high fidelity to the intervention protocol. This also mirrors the high therapist acceptance of the OT^ee^ HORSPLAY intervention (27), and indicates that the modifications to the OT-ZOR Equine protocol successfully increased standardization without losing the client-centered individualization that is essential to occupational therapy practice (57).

Preliminary participant outcomes suggest that self-regulation indicators improved after OT-ZOR Clinic and plateaued after OT-ZOR Equine, but we do not know if participants would have demonstrated the same improvements had they participated in OT-ZOR Equine first. Preliminary participant outcomes also suggest that OT-ZOR Equine may offer additive benefits in social functioning, particularly social communication, compared to OT-ZOR Clinic. These findings are consistent with social communication improvements seen after several different equine-assisted services for autistic youth (58).

### 4.1 Limitations

This study occurred in 2021 as the US was emerging from the COVID-19 pandemic, so school re-openings, masking, and a variety of other historical factors likely affected the outcomes. The study is also limited by a small, non-diverse sample and reliance on parent-report outcome measures, which could be biased by caregivers’ investment in the therapy process. Finally, most caregivers enrolled in the study were youths’ mothers; therefore, this study does not represent fathers’ views on the acceptability or preliminary outcomes of the intervention, which may differ from mothers’ perspectives.

The current study was designed to assess *feasibility* of OT-ZOR Equine, and we are limited in our ability to draw efficacy conclusions due to possible order effects, attention effects, or the effect of the time of year on outcomes. Since all participants received OT-ZOR Clinic first, followed by OT-ZOR Equine, we are unable to draw efficacy conclusions about OT-ZOR Equine alone. Historical effects are also of particular concern, given data collection occurred in 2021 as the US was emerging from the COVID-19 pandemic. For instance, significant improvements in caregiver physical quality of life may have been impacted by the receding COVID-19 pandemic (e.g., questions such as “how satisfied are you with your capacity for work?” may have been impacted by easing social distancing and remote-work requirements). Further, school re-openings with masking requirements may have affected the lack of improvement in participants’ social motivation. Notwithstanding, these promising preliminary participant outcomes suggest the need for a larger randomized controlled trial that randomly assigns participants to receive either OT-ZOR Equine or OT-ZOR Clinic to assess the additive benefit of integrating horses into occupational therapy on self-regulation and social outcomes in autistic youth. In future efficacy studies researchers should recruit a larger diverse sample, randomly assign participants to conditions, and include physiological indicators of self-regulation in the outcome battery.

### 4.2 Conclusion

OT-ZOR Equine is feasible to implement, acceptable to recipients’ caregivers and providing occupational therapists, and may offer additive improvements in social functioning compared to occupational therapy without horses. This study provides a strong foundation for a future randomized controlled trial to assess the efficacy of integrating horses into occupational therapy to improve self-regulation and social outcomes in autistic youth.

## Data Availability

The raw data supporting the conclusions of this article will be made available by the authors, without undue reservation.
